# Supplementary material for: Evolution of genome structure in the Drosophila simulans species complex
Source: Genome Res. 2021 Mar;31(3):380–96. doi: 10.1101/gr.263442.120 (PMC7919458; doi:10.1101/gr.263442.120)
Supplement: Supplemental Material [file supp_31_3_380__index.html]

Evolution of genome structure in the Drosophila simulans species complex — Supplemental Material 

# Evolution of genome structure in the *Drosophila simulans* species complex

## Supplemental Material

- supplemental\_Table\_S18.xlsx
- supplemental\_Table\_S17.xlsx
- supplemental\_Table\_S16.xlsx
- supplemental\_Table\_S15.xlsx
- supplemental\_Table\_S14.xlsx
- supplemental\_Table\_S13.xlsx
- supplemental\_Table\_S12.xls
- supplemental\_Table\_S11.xlsx
- supplemental\_Table\_S10.xlsx
- supplemental\_Table\_S9.xlsx
- supplemental\_Table\_S8.xlsx
- supplemental\_Table\_S7.xlsx
- supplemental\_Table\_S6.xlsx
- supplemental\_Table\_S5.xlsx
- supplemental\_Table\_S4.xlsx
- supplemental\_Table\_S3.xlsx
- supplemental\_Table\_S2.xlsx
- supplemental\_Table\_S1.xlsx
- Supplementary\_Information\_revision3.pdf
- SupplementalFile\_S1.zip
